# Lifestyle factors in Black female breast cancer survivors—Descriptive results from an online pilot study

**DOI:** 10.3389/fpubh.2023.1072741

**Published:** 2023-03-14

**Authors:** Avonne E. Connor, Kate E. Dibble, Kala Visvanathan

**Affiliations:** ^1^Johns Hopkins Bloomberg School of Public Health, Baltimore, MD, United States; ^2^Johns Hopkins Sidney Kimmel Comprehensive Cancer Center, Baltimore, MD, United States; ^3^Dana-Farber Cancer Center, Boston, MA, United States

**Keywords:** breast cancer, survivorship, Black women, social networks (online), lifestyle factors

## Abstract

**Background:**

Breast cancer (BC) research examining disparities in cancer survivorship and modifiable risk behaviors has been mostly cancer-specific, leaving relevant gaps in disparities research relating to other cancer survivorship outcomes, including cardiovascular disease (CVD). Maintaining healthy lifestyle behaviors is a critical component of successful cancer survivorship, where unhealthy behaviors may increase the risk for recurrence, second primary cancers, and incidence of new comorbid conditions, including CVD. The current study describes BC survivorship factors among an online pilot study of Black BC survivors in Maryland, with a focus on the burden of obesity, comorbidity, and behavioral factors associated with CVD risk.

**Methods:**

Utilizing social media recruitment strategies and survivor networks, we recruited 100 Black female BC survivors to complete an online survey. Descriptive characteristics (demographic, clinical, and lifestyle factors) were analyzed in terms of frequencies, means, standard deviations (SD) overall and by county.

**Results:**

The average ages at time of survey and at primary BC diagnosis were 58.6 years (*SD* = 10.1) and 49.1 years (*SD* = 10.2), respectively. More than half of the survivors reported having hypertension (51%); and while only 7% reported being obese at the time of BC diagnosis, 54% reported being obese at the time of survey which was on average 9 years post BC diagnosis. Only 28% of the survivors reported meeting weekly exercise recommendations. While 70% were never smokers, most ever smokers resided in Baltimore City/Baltimore County (*n* = 18 ever smokers).

**Conclusion:**

Our pilot study identified at-risk BC survivors in Maryland due to the high prevalence of CVD risk factors (hypertension, obesity, limited exercise). These pilot study methods will inform a future statewide multilevel prospective study to improve health behaviors among Black BC survivors.

## Introduction

African American/Black female breast cancer patients have the highest breast cancer mortality rates and shortest overall survival than any racial/ethnic group of women in the US (1). When diagnosed with breast cancer, Black women are diagnosed at younger ages, with more advanced stages, and have a higher prevalence of more aggressive breast cancer subtypes that can grow rapidly with a worse prognosis compared to non-Hispanic white women with the same tumor factors (2). Black breast cancer patients also experience significant healthcare system-related disparities including delays in diagnostic and therapeutic care (3–5) and insufficient knowledge of cancer follow-up care (6).

The cause of these disparities in breast cancer and overall survival among Black women is multifactorial and reflects the interplay between biological factors (differences in tumor biology, advanced stage of disease, individual health status) and socioeconomic and societal disparities. The high prevalence of obesity and obesity-associated comorbid conditions can impact late stage of presentation, cancer progression, choice of treatment, and overall survival (7–11). Unequal access to opportunities and resources such as wealth, income, and education can also influence access to high-quality healthcare services, such as health education, disease prevention/screening, early detection, and treatment services (1) and can directly affect health outcomes and risk factors associated with poor prognosis (12). Independent of socioeconomic status, minority cancer patients might also be exposed to additional stressors associated with forms of discrimination and racism which can impact health outcomes throughout life (13). The persistent exposures of these factors together, socioeconomic adversity, racism, and discrimination, have been coined the term “weathering” which was specifically hypothesized by Geronimus to affect the health of Black women (14).

Partially due to these psychosocial stressors described, Black women have high rates of hypertension (50%) compared to women of other racial/ethnic groups (39% of non-Hispanic white women and 38% of Hispanic women); and furthermore, among Black women with hypertension, only 26.5% have their blood pressure controlled (15, 16). More specifically for Black breast cancer survivors, having a co-existing hypertension is very common. In a study of co-existing and newly diagnosed comorbidities among breast cancer patients in Missouri, we found that Black patients were 1.4 times more likely to have a diagnosis of hypertension (53%) compared to White patients (17). Hypertension and obesity were also found to be highly prevalent among young Black breast cancer patients with triple-negative breast cancer (18).

Co-existing comorbidities can increase risk of breast cancer mortality by 20–50% and competing-cause mortality up to six-fold (19). While the risk of cardiovascular disease (CVD) mortality following breast cancer has been studied (20–22), few studies have examined these associations among Black women (23, 24), who have a high prevalence of CVD risk factors at time of cancer diagnosis. Among a study of 25,181 White and 10,907 Black female breast cancer patients identified from the Maryland Cancer Registry, we observed a 33% increased risk of CVD mortality among Black women compared to White women, with almost 3-fold increased hazard of CVD mortality among women 50–59 years of age in age-stratified results (*p*-int. < 0.01) (25). A notable limitation of our study was that we lacked data on important CVD risk factors that can be treated and modified, such as obesity, hypertension, and behavioral lifestyle factors.

As a next step to address this limitation and to acquire a better understanding of the comorbidity burden and prevalence of behavioral risk factors associated with CVD risk among Black breast cancer survivors in Maryland, we conducted a pilot study of Black breast cancer survivors living in Maryland using social media recruitment strategies. Our team piloted study methods to inform a future community-based intervention study of Black breast cancer survivors at high risk of poor outcomes due to comorbid conditions and social inequities (26). Maryland is a significant resource to research breast cancer disparities due to its racial and socioeconomic diversity (27), particularly by county of residence as Prince George's County has one of the highest median household incomes in the US according to 2019 data ($84,920 compared to Baltimore City with a median household income of $50,379) (28). The current pilot study describes breast cancer survivorship factors and the burden of obesity, comorbidity, and behavioral factors associated with CVD risk among a sample of Black breast cancer survivors across the state and explores differences by county to determine if potential societal differences may exist.

## Methods

### Study design and population

Detailed recruitment methods of this cross-sectional study have been previously described (26). Recruitment from social media platform click-oriented ads *via* Meta and Instagram began on January 5, 2022 and completed on August 18, 2022. After ~6 months of recruitment, our Facebook ad reached 118,461 individuals on Facebook and 2,650 link clicks, had 3,539 post engagements, 611 post reactions, and 181 post shares. A total of 127 women completed the online screener and consented to participate, and of those women, 100 completed the online survey. All participants had to identify as biologically female, African American/Black, have been diagnosed with breast cancer (stages *in situ*-IV), and reside in Maryland (identified by geolocation).

Social media accounts were collected but were utilized for ad purposes, as there was only direct contact between participant (or potential participant) and research team through Facebook study “Pages” direct messaging and *via* institutional email addresses. By private messaging through the “Pages” forum, research administration could view names and information (depending on participants' Meta and Instagram privacy settings) without research administration using their own social media accounts. Therefore, they were directly conversing with participants *via* a “Page” front. This study was approved by the Johns Hopkins Bloomberg School of Public Health Institutional Review Board (IRB #00018654).

Recruitment took place using an online, anonymous eligibility screening mechanism *via* REDCap^©^ (29) managed by the Johns Hopkins University Information Technology department. Individuals who met inclusion criteria for the current study were then redirected to the electronic consent page to review passive consent information and by clicking the “next” button they provide consent to participate in the full online survey. Eligible participants provided their email address for compensation and recontact purposes (regarding future contact for new studies). While most participants found the study information through the Facebook advertisement, some participants were also recruited directly from their social networks (friend referrals), including local cancer survivorship support groups. Of note, some of the participants did not have Facebook accounts. Upon completion of the online survey, participants were emailed a $25 Amazon e-gift card to the email address provided.

### Online survey

The online survey was administered *via* REDCap^©^ and was designed to take ~30 min to complete. Survey sections focused on several factors including: demographic characteristics (current age, age at breast cancer primary diagnosis, state of residence, county of residence, annual income, education, employment), clinical characteristics (cancer diagnoses, stage at breast cancer diagnosis, cancer treatments, recurrences, metastases), recent and former cancer screening history [mammography, magnetic resonance imaging (MRI)], genetic testing information (if they completed genetic testing), health promotion questions [weight history, smoking, alcohol consumption, diet/nutrition, physical activity (PA)], comorbid conditions, COVID-19 impact, healthcare access and utilization, and quality of physician-patient communication (treatment decisions, sufficient time, understanding, respectful, genuine, available treatment options, etc.). The online survey also collected preferences for future interventions, willingness to participate in medical record abstraction, and email addresses for compensation and future contact.

### Variables of interest

The following variables were of interest for the current analysis: demographic factors (current age, age at primary breast cancer diagnosis, county of residence, annual income, marital status, health insurance status, education, employment), clinical characteristics (cancer diagnoses, stage of breast cancer at diagnosis, cancer treatments, recurrences, second primary diagnoses, metastases), cancer screening history, ever completed genetic testing, behavioral/lifestyle factors [weight history and height for body mass index (BMI) calculation currently, BMI at breast cancer diagnosis and BMI at age 25 years, smoking status, alcohol consumption, diet/nutrition, PA], and ever diagnosed with specific comorbid conditions. County of residence was collapsed into the following groups based on frequencies (Baltimore City/Baltimore County, Prince George's County; Other). Comorbid conditions were also grouped by the total number (calculated from ever diagnosed + breast cancer diagnosis) and then categorically: breast cancer only; 2–5 comorbid conditions; 6+ comorbid conditions. PA was determined using the Centers for Disease Control and Prevention's (CDC) National Health and Nutrition Examination Survey (NHANES) Physical Activity Questionnaire for vigorous- and moderate-intensity and strength training minutes/week, days/week, hours/day, and minutes/day types of PA. These variables were utilized to calculate minutes of each type of activity per week and on how many days per week each activity was completed. Number of days per week and minutes/week were utilized to determine if each participant had met aerobic (moderate- or vigorous-intensity PA) and/or strength training PA per week, determined by the American College of Sports Medicine (ACSM) exercise recommendations for cancer populations (30). Recommended weekly aerobic PA recommendations were met (did not meet recommendations vs. met recommendations) if participants reported at least 150 min/week of moderate-intensity or at least 75 min/week of vigorous-intensity (or a combination of both) activity on three or more days/week. Weekly strength training recommendations were met (did not meet recommendations vs. met recommendations) if the participant reported strength training PA of at least moderate-intensity on at least 2 days/week. Sedentary and light-intensity PA minutes/week were not utilized in ACSM categorizations, however, descriptive statistics were analyzed.

### Statistical analysis

Responses from the online survey were downloaded from REDCap into SAS version 9.4 and Stata version 16. Descriptive characteristics (demographic, clinical, and lifestyle factors) were analyzed in terms of frequencies, means, standard deviations (SD) overall and by county. Statistical analyses were performed using Stata version 16.

## Results

Table 1 shows the demographic characteristics of the study population (*n* = 100 completed surveys) overall and by county of residence. The average ages at time of survey and at primary breast cancer diagnosis were 58.6 years (*SD* = 10.1) and 49.1 years (*SD* = 10.2), respectively, with an average of 9.2 years (*SD* = 8.4) between primary breast cancer diagnosis and time of survey. Women reported living in Prince George's County (33%) and Baltimore City/Baltimore County (33%) Maryland, and other counties (34%). For sociodemographic factors, 36% reported having a college degree as their highest level of education, and 47.1% have an annual household income of ≥$75,000. When asked about participation in future research studies, 99% consented to be contacted in the future and 79% reported they would be interested in having their medical records abstracted for research purposes (data not shown).

**Table 1:** Demographic characteristics of study population overall and stratified by Maryland county (*N* = 100).

	**Total (*N* = 100) *N* (%)**	**Maryland County**		
		**Baltimore City/County (*****N*** = **33)** ***N*** **(%)**	**Prince George's (*****N*** = **33)** ***N*** **(%)**	**Other (*****N*** = **34)** ***N*** **(%)**
**Education**				
High school/GED or less	13 (13.0)	7 (21.2)	3 (9.1)	3 (8.8)
Some college	23 (23.0)	9 (27.3)	5 (15.2)	9 (26.5)
4-year college degree	36 (36.0)	12 (36.4)	16 (48.5)	8 (23.5)
Any graduate degree	28 (28.0)	5 (15.2)	9 (27.3)	14 (41.2)
Missing	0 (0.0)	0 (0.0)	0 (0.0)	0 (0.0)
**Current marital status**				
Married or living as married	38 (38.0)	8 (24.2)	11 (33.3)	19 (55.9)
Divorced	24 (24.0)	7 (21.2)	9 (27.3)	8 (23.5)
Separated	3 (3.0)	1 (3.0)	0 (0.0)	2 (5.9)
Widowed	5 (5.0)	4 (12.1)	0 (0.0)	1 (2.9)
Single (never married)	29 (29.0)	12 (36.4)	13 (39.4)	4 (11.8)
Missing	1 (1.0)	1 (3.0)	0 (0.0)	0 (0.0)
**Current employment status**				
Working full-time	45 (45.0)	14 (42.4)	13 (39.4)	18 (52.9)
Retired	31 (31.0)	14 (42.4)	8 (24.2)	9 (26.5)
Other (disability, unemployed, homemaker, working part-time)	24 (24.0)	5 (15.2)	12 (36.4)	7 (20.6)
Missing	0 (0.0)	0 (0.0)	0 (0.0)	0 (0.0)
**Annual household income**				
Less than $40,000	24 (24.0)	9 (27.3)	8 (24.2)	7 (20.6)
$40,000–75,000	26 (26.0)	17 (51.5)	6 (18.2)	3 (8.8)
$75,000–99,000	21 (21.0)	3 (9.1)	7 (21.2)	11 (32.4)
$100,000 or more	26 (26.0)	3 (9.1)	11 (33.3)	12 (35.3)
Missing	3 (3.0)	1 (3.0)	1 (3.0)	1 (2.9)
**Health insurance at diagnosis**				
No insurance	2 (2.0)	0 (0.0)	1 (3.0)	1 (2.9)
Private	69 (69.0)	22 (66.7)	23 (69.7)	24 (70.6)
Medicare or Medicaid	16 (16.0)	5 (15.2)	4 (12.1)	7 (20.6)
Other	13 (13.0)	6 (18.2)	5 (15.2)	2 (5.9)
Missing	0 (0.0)	0 (0.0)	0 (0.0)	0 (0.0)
**Health insurance currently**				
No insurance	0 (0.0)	0 (0.0)	0 (0.0)	0 (0.0)
Private	50 (50.0)	12 (36.4)	18 (54.5)	20 (58.8)
Medicare or Medicaid	36 (36.0)	15 (45.5)	9 (27.3)	12 (35.3)
Other	13 (13.0)	5 (15.2)	6 (18.2)	2 (5.9)
Missing	1 (1.0)	1 (3.0)	0 (0.0)	0 (0.0)
**Residency status**				
Homeowner	63 (63.0)	19 (57.6)	22 (66.7)	22 (64.7)
Not a homeowner	37 (37.0)	14 (42.4)	11 (33.3)	12 (35.3)
Missing	0 (0.0)	0 (0.0)	0 (0.0)	0 (0.0)
	***M*** **(*****SD*****) Range**	***M*** **(*****SD*****) Range**	***M*** **(*****SD*****) Range**	***M*** **(*****SD*****) Range**
Current age (in years)	58.6 (10.1) 32.0–78.0	62.4 (8.63) 41.0–76.0	58.0 (9.29) 33.0–78.0	55.3 (11.2) 32.0–71.0
Age at primary breast cancer diagnosis (in years)	49.1 (10.2) 25.0–78.0	47.7 (9.99)26.0–64.0	49.9 (10.3) 25.0–78.0	49.6 (10.5) 28.0–69.0

Table 2 shows the clinical characteristics of the study population overall and by county. Most women (70%) were diagnosed with stages I-IV BC and only 22% were currently undergoing treatment at the time of the survey. A total of 19 women reported experiencing a breast cancer recurrence, 15% reported a metastasis, and 15% also reported experiencing a second primary cancer. A total of 60 women reported having genetic testing and 32 had first-degree relatives with breast cancer. While 77% reported having breast cancer screening in the past year, the lowest prevalence of recent breast cancer screening was observed among survivors living in Baltimore City/Baltimore County (60.6%).

**Table 2:** Clinical cancer characteristics of the study population overall and stratified by Maryland county (*N* = 100).

	**Total (*N* = 100) *N* (%)**	**Maryland County**		
		**Baltimore City/County (*****N*** = **33)** ***N*** **(%)**	**Prince George's (*****N*** = **33)** ***N*** **(%)**	**Other (*****N*** = **34)*****N*** **(%)**
**Stage of breast cancer at diagnosis**				
*In situ*	28 (28.0)	11 (33.3)	9 (27.3)	8 (23.5)
Stages I-IV	70 (70.0)	21 (63.6)	23 (69.7)	26 (76.5)
Missing	2 (2.0)	1 (3.0)	1 (3.0)	0 (0.0)
**Previous breast cancer treatment completed**				
Breast-conserving surgery	51 (51.0)	18 (54.5)	16 (48.5)	17 (50.0)
Mastectomy	46 (46.0)	17 (51.5)	14 (42.4)	15 (44.1)
Radiation	58 (58.0)	18 (54.5)	22 (66.7)	18 (52.9)
Adjuvant chemotherapy	36 (36.0)	16 (48.5)	11 (33.3)	9 (26.5)
Neoadjuvant chemotherapy	14 (14.0)	1 (3.0)	6 (18.2)	7 (20.6)
Hormone or endocrine therapy	47 (47.0)	13 (39.4)	13 (39.4)	21 (61.8)
Targeted drug therapy	6 (6.0)	0 (0.0)	3 (9.1)	3 (9.1)
Immunotherapy or biological therapies	3 (3.0)	0 (0.0)	1 (3.0)	2 (5.9)
Stem cell transplant	0 (0.0)	0 (0.0)	0 (0.0)	0 (0.0)
Precision medicine	1 (1.0)	0 (0.0)	0 (0.0)	1 (2.9)
Clinical trial	4 (4.0)	1 (3.0)	1 (3.0)	2 (5.9)
No treatment(s)	3 (3.0)	0 (0.0)	2 (6.1)	1 (2.9)
**Current status of cancer treatment**				
Currently undergoing treatment	22 (22.0)	3 (9.1)	9 (27.3)	10 (29.4)
Completed treatment	69 (69.0)	26 (78.8)	22 (66.7)	21 (61.8)
Did not receive treatment	5 (5.0)	2 (6.1)	2 (6.1)	1 (2.9)
Missing	4 (4.0)	2 (6.1)	0 (0.0)	2 (5.9)
**Experienced a recurrence**				
No	80 (80.0)	25 (75.8)	27 (81.8)	28 (82.4)
Yes	19 (19.0)	8 (24.2)	5 (15.2)	6 (17.6)
Missing	1 (1.0)	0 (0.0)	1 (3.0)	0 (0.0)
**Metastases in breast cancer diagnosis**				
No	85 (85.0)	28 (84.8)	27 (81.8)	30 (88.2)
Yes	15 (15.0)	5 (15.2)	6 (18.2)	4 (11.8)
Missing	0 (0.0)	0 (0.0)	0 (0.0)	0 (0.0)
**Experienced second primary**				
No	84 (84.0)	27 (81.8)	29 (85.3)	28 (84.8)
Yes	15 (15.0)	6 (18.2)	5 (14.7)	4 (12.1)
Missing	1 (1.0)	0 (0.0)	0 (0.0)	1 (3.0)
**Experienced any other cancer diagnoses in lifetime**				
No	85 (85.0)	27 (81.8)	27 (81.8)	31 (91.2)
Yes	15 (15.0)	6 (18.2)	6 (18.2)	3 (8.8)
Missing	0 (0.0)	0 (0.0)	0 (0.0)	0 (0.0)
**Completed breast cancer screening measured in the last year**				
No	22 (22.0)	13 (39.4)	3 (9.1)	6 (17.6)
Yes	77 (77.0)	20 (60.6)	29 (87.9)	28 (82.4)
Missing	1 (1.0)	0 (0.0)	1 (3.0)	0 (0.0)
**First-degree relatives with breast cancer**				
No	68 (68.0)	22 (66.7)	23 (67.6)	23 (69.7)
Yes	32 (32.0)	11 (33.3)	11 (32.4)	10 (30.3)
Missing	0 (0.0)	0 (0.0)	0 (0.0)	0 (0.0)
**Undergo genetic testing for breast cancer-related genetic mutations**				
No	40 (40.0)	18 (54.5)	11 (32.4)	11 (33.3)
Yes	60 (60.0)	15 (45.5)	23 (67.6)	22 (66.7)
Missing	0 (0.0)	0 (0.0)	0 (0.0)	0 (0.0)

Tables 3, 4 show the prevalence of comorbidities among the study population and the number of survivors with multiple chronic conditions/multimorbidity, respectively. More than half of the study participants reported having hypertension (51%, of which most were taking hypertension medication *n* = 43), followed by arthritis (45%), lymphedema (32%) and self-reported obesity (32%) (Table 3), and 28% reported having ≥6 comorbid conditions (Table 4). General health perceptions were mostly good to excellent (79%); however, 28% reported worse health currently than at the time of breast cancer diagnosis (data not shown).

**Table 3:** Comorbid conditions reported by study participants overall and by Maryland county.

	**Ever diagnosed** ***N*** **(%)**	**Maryland County**		
		**Baltimore City/County (*****N*** = **33)** ***N*** **(%)**	**Prince George's (*****N*** = **33)** ***N*** **(%)**	**Other (*****N*** = **34)** ***N*** **(%)**
**Comorbid conditions**				
Hypertension	51 (51.0)	21 (63.6)	14 (42.4)	16 (47.1)
Arthritis	45 (45.0)	17 (51.5)	19 (57.6)	9 (26.5)
Lymphedema	32 (32.0)	14 (42.2)	8 (24.2)	10 (29.4)
Obesity	32 (32.0)	8 (24.2)	9 (27.3)	15 (44.1)
Anemia	27 (27.0)	8 (24.2)	10 (30.3)	9 (26.5)
Anxiety	25 (25.0)	9 (27.3)	3 (9.1)	13 (38.2)
Diabetes	23 (23.0)	8 (24.2)	7 (21.2)	8 (23.5)
Depression	20 (20.0)	8 (24.2)	3 (9.1)	9 (26.5)
Gastrointestinal issues or GERD	19 (19.0)	8 (24.2)	4 (12.1)	7 (20.6)
Peripheral neuropathy	17 (17.0)	5 (15.2)	6 (18.2)	6 (17.6)
Hypothyroidism/hyperthyroidism	13 (13.0)	4 (12.1)	5 (15.2)	4 (11.8)
Asthma	11 (11.0)	6 (18.2)	2 (6.1)	3 (8.8)
Osteopenia/osteoporosis	8 (8.0)	4 (12.1)	4 (12.1)	0 (0.0)
Kidney disease	7 (7.0)	5 (15.2)	0 (0.0)	2 (5.9)
Blood clots	6 (6.0)	2 (6.1)	3 (9.1)	1 (2.9)
Other diagnoses	5 (5.0)	2 (6.1)	1 (3.0)	2 (5.9)
Hypercholesteremia	5 (5.0)	3 (9.1)	2 (6.1)	0 (0.0)
Vascular issues	4 (4.0)	2 (6.1)	2 (6.1)	0 (0.0)
Infertility	4 (4.0)	1 (3.0)	2 (6.1)	1 (2.9)
Epilepsy	3 (3.0)	1 (3.0)	0 (0.0)	2 (5.9)
Hyperlipidemia	3 (3.0)	1 (3.0)	1 (3.0)	1 (2.9)
Deep vein thrombosis	2 (2.0)	1 (3.0)	0 (0.0)	1 (2.9)
Genetic immune diseases	2 (2.0)	0 (0.0)	1 (3.0)	1 (2.9)
Stroke	2 (2.0)	1 (3.0)	0 (0.0)	1 (2.9)
HIV/AIDS	1 (1.0)	1 (3.0)	0 (0.0)	0 (0.0)
Parkinson's disease	1 (1.0)	1 (3.0)	0 (0.0)	0 (0.0)
Cardiovascular disease	0 (0.0)	0 (0.0)	0 (0.0)	0 (0.0)
Chronic pulmonary disease	0 (0.0)	0 (0.0)	0 (0.0)	0 (0.0)
Alzheimer's disease or dementia	0 (0.0)	0 (0.0)	0 (0.0)	0 (0.0)

**Table 4:** Number of comorbid conditions for the study population overall and by Maryland county.

**Number of comorbid conditions**	**Total** ***N*** = **100** ***M*** **(*****SD*****) Range**	**Maryland County**		
		**Baltimore City/County (*****N*** = **33)** ***N*** **(%)**	**Prince George's (*****N*** = **33)** ***N*** **(%)**	**Other (*****N*** = **34)** ***N*** **(%)**
Number of comorbid conditions (calculated from ever diagnosed + breast cancer diagnosis)	4.68 (2.67) 1.0–19.0	5.27 (3.54) 2.0–19.0	4.21 (1.57) 1.0–9.0	4.55 (2.50) 1.0–12.0
	***N*** **(%)**	***N*** **(%)**	***N*** **(%)**	***N*** **(%)**
**Multimorbidity**				
Breast cancer only	2 (2.0)	0 (0.0)	1 (3.0)	1 (2.9)
2–5 comorbid conditions	70 (70.0)	23 (69.7)	26 (78.8)	21 (61.8)
6+ comorbid conditions	28 (28.0)	10 (30.3)	6 (18.2)	12 (35.3)
Missing	0 (0.0)	0 (0.0)	0 (0.0)	0 (0.0)

Table 5 shows the prevalence of behavioral factors and obesity for the study population overall and by county. Of note, while only 7% reported being obese (BMI ≥ 30 kg/m^2^) at the time of the breast cancer diagnosis, 54% reported being obese at the time of their online survey. Most women (95%) reported drinking one or fewer alcoholic drinks/day and 70% were never smokers; however, most ever smoking participants resided in Baltimore City/Baltimore County (*n* = 18). Only 6 women reported current smoking overall. Only 28% of the survivors reported meeting ACSM weekly exercise recommendations of more than 150 min/week of vigorous or moderate activity and 35% reported meeting ACSM strength training recommendations weekly.

**Table 5:** Prevalence of behavioral factors and obesity for the study population overall and by Maryland county (*N* = 100).

	**Total (*N* = 100) *N* (%)**	**Maryland County**		
		**Baltimore City/County (*****N*** = **33)** ***N*** **(%)**	**Prince George's (*****N*** = **33)** ***N*** **(%)**	**Other (*****N*** = **34)** ***N*** **(%)**
**Alcohol in the past 12 months**				
Never drink alcohol	27 (27.0)	8 (24.2)	7 (21.2)	12 (35.3)
Drink alcohol	73 (73.0)	25 (75.8)	26 (78.8)	22 (64.7)
Missing	0 (0.0)	0 (0.0)	0 (0.0)	0 (0.0)
**ACS Alcohol intake recommendations**				
One or fewer drinks/day	95 (95.0)	33 (100.0)	30 (90.9)	32 (94.1)
Two or more drinks/day	5 (5.0)	0 (0.0)	3 (9.1)	2 (5.9)
Missing	0 (0.0)	0 (0.0)	0 (0.0)	0 (0.0)
**Currently smoke cigarettes**				
No	94 (94.0)	29 (87.9)	33 (100.0)	32 (94.1)
Yes	6 (6.0)	4 (12.1)	0 (0.0)	2 (5.9)
Missing	0 (0.0)	0 (0.0)	0 (0.0)	0 (0.0)
**Ever smoke cigarettes**				
No	70 (70.0)	15 (45.5)	27 (81.8)	28 (82.4)
Yes	30 (30.0)	18 (54.5)	6 (18.2)	6 (17.6)
Missing	0 (0.0)	0 (0.0)	0 (0.0)	0 (0.0)
**Overall health of diet**				
Excellent	11 (11.0)	4 (12.1)	3 (9.1)	4 (11.8)
Very good	18 (18.0)	5 (15.2)	7 (21.2)	6 (17.6)
Good	32 (32.0)	11 (33.3)	14 (42.4)	7 (20.6)
Fair	29 (29.0)	11 (33.3)	5 (15.2)	13 (38.2)
Poor	3 (3.0)	0 (0.0)	1 (3.0)	2 (5.9)
Missing	7 (7.0)	2 (6.1)	3 (9.1)	2 (5.9)
**Obesity status currently**				
Underweight/normal weight (BMI < 24.9)	10 (10.0)	1 (3.0)	7 (21.2)	2 (5.9)
Overweight (BMI 25.0 to < 29.9)	34 (34.0)	13 (39.4)	9 (27.3)	12 (35.3)
Obese (BMI ≥ 30)	54 (54.0)	18 (54.5)	17 (51.5)	19 (55.9)
Missing	2 (2.0)	1 (3.0)	0 (0.0)	1 (2.9)
**Obesity status at age 25**				
Underweight/normal weight (BMI < 24.9)	53 (53.0)	20 (60.6)	16 (48.5)	17 (50.0)
Overweight (BMI 25.0 to < 29.9)	23 (23.0)	7 (21.2)	9 (27.3)	7 (20.6)
Obese (BMI ≥ 30)	14 (14.0)	5 (15.2)	5 (15.2)	4 (11.8)
Missing	10 (10.0)	1 (3.0)	3 (9.1)	6 (17.6)
**Obesity status at diagnosis**				
Underweight/normal weight (BMI < 24.9)	74 (74.0)	21 (63.6)	26 (78.8)	27 (79.4)
Overweight (BMI 25.0 to < 29.9)	13 (13.0)	6 (18.2)	5 (15.2)	2 (5.9)
Obese (BMI ≥ 30)	7 (7.0)	3 (9.1)	2 (6.1)	2 (5.9)
Missing	6 (6.0)	3 (9.1)	0 (0.0)	3 (8.8)
**Meeting ACSM weekly aerobic PA recommendations (**>**150 min/wk vigorous or moderate activity)**				
Does not meet recommendations	56 (56.0)	18 (54.5)	19 (57.6)	19 (55.9)
Meets aerobic recommendations	28 (28.0)	12 (36.4)	8 (24.2)	8 (23.5)
Missing	16 (16.0)	3 (9.1)	6 (18.2)	7 (20.6)
**Meets ACSM weekly strength training PA recommendations (2**×**per week)**				
Does not meet recommendations	51 (51.0)	19 (57.6)	17 (51.5)	15 (44.1)
Meets aerobic recommendations	35 (35.0)	10 (30.3)	12 (36.4)	13 (38.2)
Missing	14 (14.0)	4 (12.1)	4 (12.1)	6 (17.6)
	***M*** **(*****SD*****) Range**	***M*** **(*****SD*****) Range**	***M*** **(*****SD*****) Range**	***M*** **(*****SD*****) Range**
Current body mass index (BMI)	31.7 (6.95) 19.7–63.0	32.5 (5.98) 23.3–46.1	30.6 (8.61) 19.7–63.0	32.0 (5.95) 20.9–43.8
BMI at age 25	24.9 (5.06) 15.6–46.8	24.1 (4.32) 15.6–32.2	25.4 (5.64) 17.2–46.8	25.2 (5.25) 18.1–39.4
BMI at diagnosis	21.0 (6.18) 10.1–42.4	21.7 (6.47) 13.6–38.0	21.4 (6.74) 10.1–42.4	20.0 (5.25) 11.7–36.3
Vigorous PA—min/wk	102.0 (225.2) 0.0–1,220.0	129.8 (273.5) 0.0–1,220.0	104.6 (238.8) 0.0–854.0	66.2 (131.2) 0.0–427.0
Moderate PA—min/wk	301.5 (541.1) 0.0–2,520.0	317.7 (589.7) 0.0–2,520.0	155.2 (244.2) 0.0–840.0	424.4 (666.9) 0.0–2,310.0
Walking PA—min/wk	454.5 (627.7) 0.0–2,940.0	480.0 (688.5) 50.0–2,940.0	333.1 (426.3) 0.0–1,680.0	538.2 (713.7) 0.0–2,520.0
Sedentary activity—min/wk	2,640.0 (1,886.7) 70.0–10,080.0	2,448.3 (1,967.8) 240.0–86,840.0	2,771.0 (1,979.6) 120.0–10,080.0	2,704.8 (1,748.2) 70.0–8,400.0
Aerobic (Vig+Mod PA)—min/wk	385.7 (713.8) 0.0–3,740.0	442.5 (858.4) 0.0–3,740.0	270.3 (447.1) 0.0–1,389.0	453.1 (790.6) 0.0–2,615.0
Strength training—min/wk	89.6 (136.1)	68.6 (104.2) 0.0–420.0	100.0 (153.0) 0.0–630.0	100.5 (148.6) 0.0–630.0
Age began smoking cigarettes	18.2 (7.85) 12.0–50.0	17.9 (6.06) 12.0–35.0	15.1 (2.31) 12.0–18.0	22.0 (13.9) 12.0–50.0
Time since quitting smoking cigarettes (years)	20.5 (14.9) 0.0–50.0	22.7 (15.0) 0.0–50.0	20.5 (10.6) 10.0–38.0	10.6 (19.6) 0.4–40.0
When quit, # of cigarettes smoked daily	9.50 (9.55) 0.0–40.0	9.75 (10.3) 0.0–40.0	13.50 (8.57) 2.0–23.0	2.50 (1.00) 1.0–3.0
Past 30 days on the days smoked, how many cigarettes smoked	2.65 (3.06) 0.0–10.0	3.08 (3.08) 0.0–10.0	1.67 (2.16) 0.0–6.0	2.80 (4.20) 0.0–10.0

ACSM, American College of Sports Medicine; PA, physical activity.

## Discussion

Our pilot study examined a new recruitment strategy that could increase the enrollment and retention of minority breast cancer survivors into community-based studies. Maryland is the ideal state to conduct such a study given that the African American/Black population represents more than 30% of the total population, unlike the US where the African American/Black population is close to 14% (27). For initial study findings, we have identified at-risk breast cancer survivors in Maryland due to high prevalence of CVD risk factors including high prevalence of hypertension, current obesity due to weight gain post diagnosis, and lack of meeting PA recommendations.

Hypertension is the most prevalent comorbidity reported among breast cancer patients, regardless of age, followed by CVD and type-2 diabetes (31, 32). While we observed hypertension to be the most reported comorbidity among our study population, interestingly none of our study participants reported a history of CVD. Hypertension is an important comorbidity to study among Black women with breast cancer, as it has been found to account for 30% of the Black-White racial disparity in all-cause mortality (33). The timing of when hypertension develops (before or after breast cancer diagnosis) should also be considered in studies of survival disparities to determine if interventions to prevent hypertension and/or decrease its severity could be beneficial to decreasing risk of poor health outcomes. Among Black women diagnosed with invasive breast cancer in Missouri (*n* = 3,039), we identified a significant difference in risk of CVD mortality among women with a diagnosis of hypertension within 2 years of breast cancer diagnosis, in comparison with women without hypertension (subdistribution hazard ratio, 1.87; 95% confidence interval: 1.02–3.44) (17). While our current pilot study did not account for when hypertension was diagnosed, our future studies will include the timing of comorbidity diagnoses as some may be related to receipt of certain cancer treatment associated with risk of cardiac toxicity (23, 34–38) which has also been found to be prevalent among Black breast cancer patients (39, 40).

Cancer research examining disparities in cancer survivorship and modifiable risk behaviors has been mostly cancer-specific, leaving relevant gaps in disparities research relating to other cancer survivorship outcomes, including CVD outcomes. Maintaining healthy lifestyle behaviors is a critical component of successful cancer survivorship, where unhealthy behaviors may increase the risk for recurrence, second primary cancers, and incidence of new comorbid conditions, including CVD and impact quality of life post-cancer (41). A systematic literature review conducted by Tollosa et al. described health behaviors of cancer survivors, finding that the recommended health behaviors most frequently adhered to were smoking cessation and low (or no) alcohol intake (42). Also comparable with our results, research has documented that Black breast cancer survivors report higher prevalence of obesity (43, 44). Our finding that women became obese after diagnosis should be further explored. We also recently examined racial/ethnic disparities in maintaining healthy lifestyle behaviors among 2,044 female cancer survivors utilizing data from the NHANES, with almost 30% of the sample being breast cancer survivors and 14% of the sample were non-Hispanic Black (NHB) (45). Overall, NHB survivors were more likely to be overweight/obese and were more likely to report not meeting weekly exercise recommendations compared to NHW survivors. NHB survivors were also less likely to report ever smoking and were less likely to drink two or more alcoholic drinks per day, compared to NHW survivors (45).

The current analysis has strengths and limitations. Past literature on the use of social media among breast cancer survivors has shown that limited research has focused on non-Caucasian populations (46). Our study adds to the growing body of literature focused on the use of social media and networks for recruiting and conducting population-based studies among African American/Black breast cancer survivors. There are study limitations associated with social media study recruitment methods which can introduce biases. Social media recruitment has shown to capture geographic diversity, but could attract a more highly distressed subgroup with online activity that connects to patient groups and/or online activity makes them targets for recruitment ads (47). These factors could introduce selection bias with respect to lifestyle factors and comorbidity burden. For example, we observed that none of our study participants reported a history of CVD, one of the most reported comorbidities among breast cancer survivors. This finding could be attributed to a healthy survivor bias. Furthermore, social media recruitment has had limited participation of older adults (48). With comparison of our previous study of newly diagnosed breast cancer patients in Maryland utilizing Maryland Cancer Registry data, the mean age at diagnosis was 58.2 years among Black women diagnosed between 2007 and 2017 (25), while the mean age at diagnosis for the current pilot study was 49.1 years.

We also must consider there could be differences in participation by time since diagnosis, as we did not limit our inclusion criteria by active treatment or time since breast cancer diagnosis. Women who are sicker due to their cancer and/or on active treatment may have been less likely to participate and among those who participated, may have different lifestyle factors and healthcare experiences. Additionally, with respect to collection of data for lifestyle factors, it should be noted that behavioral factors showed the highest prevalence of missing values, which limits the interpretation of this data. Lastly, most of our study population reported both higher incomes and education levels and were from more urban areas. Taking into account these study limitations, our descriptive results may not be generalizable for most Black breast cancer patients and survivors in Maryland; however, our future research studies utilizing this recruitment approach will address these study limitations.

## Conclusion

Our research goal was to utilize our pilot study of community-based Black breast cancer survivors to gain a better understanding of the comorbidity burden and describe the prevalence of behavioral risk factors associated with CVD risk among Black survivors in Maryland. We identified at-risk breast cancer survivors due to the high prevalence of CVD risk factors including hypertension, current obesity, and the lack of meeting aerobic and strength training PA recommendations. These research methods will inform a statewide multilevel prospective population-based study to improve health behaviors and disease management among Black women breast cancer survivors in Maryland at high risk of poor outcomes due to biological differences and socioeconomic inequities.

## Data availability statement

The raw data supporting the conclusions of this article will be made available by the authors, without undue reservation.

## Ethics statement

The studies involving human participants were reviewed and approved by Johns Hopkins Bloomberg School of Public Health Institutional Review Board. Passive consent for participation was obtained electronically from the participants. Written informed consent for participation was not required for this study in accordance with the national legislation and the institutional requirements.

## Author contributions

AC was responsible for the study design, design of the analysis, and manuscript writing. KD was responsible for the data analysis, tables, and manuscript writing. KV was responsible for reviewing and editing the manuscript. All authors reviewed the manuscript. All authors contributed to the article and approved the submitted version.
